# Close Space Sublimation Growth of Sb_2_(S,Se)_3_ Thin-Film Solar Cells

**DOI:** 10.1021/acsami.5c10627

**Published:** 2025-10-08

**Authors:** Daniya A. Sindi, Thomas P. Shalvey, Matthew. J. Smiles, Tim. D. Veal, Leon Bowen, Jonathan. D. Major

**Affiliations:** † Department of Physics at Stephenson Inst. Renewable Energy, 4591 University of Liverpool, Liverpool L69 7ZF, U.K.; ‡ Department of Physics, College of Science, 48058 Umm Al-Qura University, Makkah 24382, Saudi Arabia; § Department of Physics, G.J. Russell Microscopy Facility, 3057 Durham University, Durham DH1 3LE, U.K.

**Keywords:** thin film, solar cell, antimony chalcogenide, sublimation, close space sublimation

## Abstract

This study reports the synthesis and characterization of Sb_2_(S,Se)_3_ thin films for thin-film photovoltaics via a single-source close space sublimation (CSS) approach. We demonstrate power conversion efficiencies of up to 4.3% with notable improvements in open-circuit voltage (*V*_oc_ >490 mV) compared to those of Sb_2_Se_3_ equivalents. Secondary ion mass spectrometry, X-ray diffraction, and cross-sectional scanning and transmission electron microscopies identified some issues in controlling film stoichiometry, particularly with sulfur loss, leading to oxide formation and elevated series resistance, reducing the fill factor in completed devices. We also identify clear discrepancies between the determined bandgap, sulfur content inferred from X-ray diffraction analysis, and sulfur content of the synthesized source material. We conclude that while CSS is a promising method for Sb_2_(S,Se)_3_ film fabrication, addressing sulfur loss during deposition is crucial to achieving further improvements in device efficiency.

## Graphical abstract

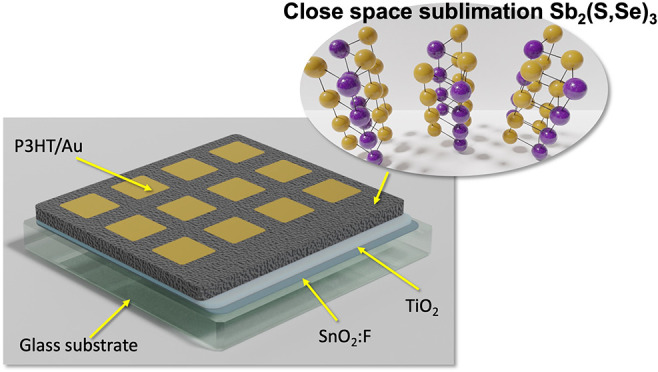

## 1 Introduction

Antimony sulfoselenide, Sb_2_(S,Se)_3_, is a promising material for thin-film photovoltaics, possessing several desirable properties that make it suitable for a variety of applications. It has exceptional optical absorption properties, good stability, low toxicity, and contains no rare elements, boding well for materials availability and affordability for a long-term target of scalable production. Sb_2_(S,Se)_3_ has a high absorption coefficient of approximately (>10^5^ cm^–1^),^1,2^ with a bandgap range between 1.1 and 1.7 eV.^3^ The ratio of sulfur and selenium (S/Se) in the compound offers an opportunity to tune the bandgap compared to the pure Sb_2_Se_3_ or Sb_2_S_3_ equivalents. The chalcogen content in Sb_2_(S_1–*y*_Se_*y*_)_3_ not only significantly impacts the range of absorption, but may also modify the defect properties within the material,^4,5^ as has been observed in other technologies such as CdTe and CdTe_*x*_Se_1–*x*_.^6^

A number of papers have reported the successful fabrication of antimony sulfoselenide cells using a hydrothermal deposition method, with this approach yielding the highest recorded power conversion efficiency (PCE) to date at 10.7%.^7^ While such solution-based techniques have proved highly effective, the ultimate scalability of such techniques is questionable due to the volume of liquid chemical waste produced. In contrast, for physical vapor deposition (PVD) routes, which have a track record of scalability, there has thus far been little reported work on antimony sulfoselenide, partly due to the particular challenges of depositing a mixed chalcogenide material. There have however been some successful attempts. In 2016, Yang et al. reported Sb_2_(S,Se)_3_ deposition using in situ sulfurization of Sb_2_Se_3_ in a rapid thermal evaporation process.^8^ By varying the amount of sulfur in the source, they were able to modify the sulfur content in the final films, with an associated shift in bandgap from 1.23 to 1.42 eV, the peak device having a PCE of 5.79%. Work by Zhang et al. in 2023 used a vapor transport deposition (VTD) technique to deposit Sb_2_(S,Se)_3_ ^9^ from a mix of Sb_2_Se_3_ and Sb_2_S_3_ source materials. By modification of the deposition temperature and ratio of Sb_2_S_3_ to Sb_2_Se_3_ included in the source, they were able to vary the final composition of the Sb_2_(S,Se)**_3_** films produced. The variation in the S/Se ratio in the source material was directly reflected in the composition of the final film. XRD and Raman analysis of varying chalcogenide ratios also demonstrated that the film orientation was primarily influenced by the Sb–Se bond.

A proven scalable technique for the fabrication of high-quality thin films is the close space sublimation (CSS) method. It is in essence a close cousin of techniques such as VTD and is widely used for the fabrication of CdTe absorber layers.^10^ CSS functions by minimizing the working distance between the substrate and source, thus decreasing the vacuum requirements to maintain a sufficiently long mean-free path of sublimated materials, while maintaining comparatively high deposition rates. This enables the employment of rough vacuum (>1 × 10^–2^ mbar) or with CSS working pressures typically falling between 1 and 100 mbar of an inert gas such as nitrogen.^11^ This short substrate-to-source distance allows a high degree of material transfer, resulting in minimal waste and high deposition rates. While it has been reasonably well reported as a deposition technique for either Sb_2_Se_3_ ^12^ or Sb_2_S_3_,^13^ producing high-quality (hk1)-oriented thin films^14^ and with an efficiency of over 9% for Sb_2_Se_3_,^15^ production of the mixed Sb_2_(S,Se)_3_ material is more challenging. While mixing Sb_2_Se_3_ and Sb_2_S_3_ powders to form a source material is one possible approach, given the different melting points, 610 and 550 °C, respectively, and vapor pressures of the two compounds, controlling the relative deposition rates is problematic. K. Li et al.^16^ attempted this approach by mixing Sb_2_S_3_ and Sb_2_Se_3_ powders as a CSS source to fabricate Sb_2_(S,Se)_3_ devices and achieved a PCE of 2.70% and a cosublimation of a controlled varying molar ratio of Sb_2_Se_3_ and Sb_2_S_3_ produced on a TiO_2_ substrate to fabricate Sb_2_(S,Se)_3_ solar cells with a PCE of 9.02%.^17^ This approach was extended by H. Li et al.^18^ who incorporated an additional ball milling and sintering step of the mixed powders to form the source material yielding PCE on 5.5%. The degree of chemical consistency afforded by this approach is questionable though, leading to the potential incorporation of multiple impurities present in the binary compounds.^19^ This also applies to approaches such as that shown in a recent study by the Chen group, who produced a single-phase alloyed Sb_2_(S,Se)_3_ source material by annealing commercial Sb_2_Se_3_ powder in a double-temperature tube furnace with sulfur vapor.^18^ Fabrication of a single-crystalline source material from elemental precursor materials offers greater control over the source material content. This however requires in-house synthesis of the starting compound, as crystalline sulfoselenide material is not readily available, adding an extra layer of challenge. In this work, we focus on CSS deposition from a single Sb_2_(S,Se)_3_ source, using material prepared in-house from elemental precursors and incorporating Sn-doping.^20^ This is done to verify the efficacy of such an approach, to establish if the S/Se composition in the source is transferred to the thin film (i.e., the source material does not degrade upon heating), and to assess the resulting device performance. This preliminary work demonstrates the ability to produce S/Se mixed anion material with a notable bandgap shift compared to Se equivalent and with PCE of >4%. We do however identify issues related to sulfur loss within the films, resulting in the formation of oxide phases, which can compromise performance. Our work identifies that while the approach is feasible, care will be required to minimize or replace sulfur losses occurring during film deposition.

## 2 Experimental Section

### 2.1 Synthesis of Sb_2_(S,Se)_3_ Thin-Film Solar Cells

TiO_2_ thin films used as the electron transport layer (ETL) with a thickness of ∼60 nm were deposited via spin coating from a solution of titanium isopropoxide in ethanol onto Tec15 FTO-coated glass substrates. Two concentrations of 0.15 and 0.3 M were prepared inside a nitrogen-filled glovebox. Solutions were prepared by mixing 2 mL of ethanol with either 20 or 40 μL of acetic acid for 0.15 and 0.3 M, respectively. The solution was stirred at 400 rpm, followed by the addition of 90 or 180 μL of titanium isopropoxide (Sigma-Aldrich) for the 0.15 and 0.3 M, respectively, while the solutions were stirred. The solutions were left to mix for 1 h. Finally, the TiO_2_ solutions were filtered into new bottles prior to deposition to remove particulates.

Antimony sulfoselenide Sb_2_(S,Se)_3_ source material for CSS deposition was prepared by sealed ampule reaction from element precursor using a 50:50 S/Se ratio and incorporating 0.5% Sn in comparison to the Sb atom as p-type dopant.^20^ For Sb_2_(S,Se)_3_ deposition CSS conditions were varied from those developed for Sb_2_Se_3_ fabrication to reoptimize the process given the change in source material. Growth was performed in two stages with an initial “seed” layer to ensure good substrate coverage followed by a higher-temperature deposition stage to increase the grain size and improve crystallinity.^21^ The first layer was deposited at source and substrate temperatures of 440 and 350 °C, respectively, under vacuum for a range of times between 5 and 20 min. The second layer was deposited at 530 °C for the source and 490 °C for the substrate for 1–15 min deposition time in 13.33 mbar of nitrogen. After the growth was completed, the chamber was cooled under nitrogen before extracting the sample.

Poly­(3-hexylthiophene-2,5-diyl (P3HT)) was used as a back-contact hole transport interface layer (HTL) to improve contacting but also to reduce the impact of pinholes.^22^ The solution was made from 10 mg of P3HT powder (Ossila) dissolved in 1 mL of chlorobenzene (Sigma-Aldrich) in a nitrogen-filled glovebox. It was deposited by spin coating on top of the absorber layer of Sb_2_Se_3_ and Sb_2_(S,Se)_3_ devices prior to back contacting to block the pinholes in the fabricated film. 100 μL of P3HT solution was spin-coated at 1000 rpm for 10 s, followed by drying at 3000 rpm for 30 s. Thermal evaporation was used to deposit gold back contacts. Grids of Au contacts with a 0.1 cm^2^ area and a thickness of ∼50 nm were produced by thermal evaporation.

### 2.2 Characterization

Surface morphologies of the films were observed by scanning electron microscopy (SEM) using a JEOL JSM 7001F, and a 15 keV electron beam energy was used. X-ray diffraction (XRD) was carried out with monochromated Cu Kα radiation (λ = 1.5406 Å) to characterize the crystal structure of the samples using a Rigaku SmartLab system in parallel beam geometry. Secondary ion mass spectrometry (SIMS) analysis was performed by Loughborough Surface Analysis Ltd. using a Cameca 7f system. The analysis was performed using Cs^+^ primary ion bombardment and a negative secondary ion. Current–voltage (*J*–*V*) measurements were taken under AM1.5 illumination using a TS Space Systems solar simulator (class AAA). A Keithley 2400 source-measure unit recorded the output current for measurement using a bias range of −1 to +1 V. External quantum efficiency (EQE) measurements were taken using a Bentham PVE300 device calibrated with a silicon photodiode. A 300–1100 nm range was used with no white light biasing applied. An Ambios XP-200 surface profiler was used to determine the film thickness by measuring the step height across a mechanically scribed region.

Cross-sectional imaging and chemical analysis were performed by encapsulating the device in resin and capped with a mica slide, followed by sectioning into 10 mm × 10 mm samples using a wire saw. The resulting sections were subsequently prepared by lapping with a 3 μm lapping film and ion-polished using a Hitachi E-3500 ion polisher. Imaging and chemical analysis were conducted without coating under variable pressure conditions (54 Pa), using a Zeiss Sigma 300VP scanning electron microscope. The system was equipped with dual 65 mm Oxford Instruments energy-dispersive X-ray (EDX) detectors, operated at an accelerating voltage of 15 keV with a 60 μm aperture and a working distance of 8.5 mm.

The optical bandgap of the absorber was determined via optical spectroscopy using a Shimadzu Solid Spec-3700 UV–vis spectrophotometer and analysis via a Tauc analysis, for a direct bandgap, i.e., *n* = 2. This was compared with the determination of the bandgap extracted from the EQE curve. This is determined by calculating the first derivative of the EQE values and identifying the wavelength value corresponding to the negative peak in the higher-wavelength region λ_g_. The bandgap energy of the devices (*E*_g,pv_) is then determined using this value and is distinct from the optical bandgap (*E*_g.,op_) determined via an optical absorption measurement.^23^

## 3 Result and Discussion

This work was undertaken primarily to establish the overall viability of single-source CSS deposition for Sb_2_(S,Se)_3_ thin-film fabrication. One of the primary perceived challenges of this approach was the possibility of degradation of the source material during transfer, particularly via sulfur loss. Initial deposition conditions were taken from our established CSS process for Sb_2_Se_3_ devices and a device structure of FTO/TiO_2_/Sb_2_(S,Se)_3_. Composition analysis of initial films was undertaken via SIMS (Figure 1) and cross-sectional electron microscopy with EDS (Figure 2), to identify the spatial uniformity of S/Se content and any other variations in chemical composition.

**Figure 1:**
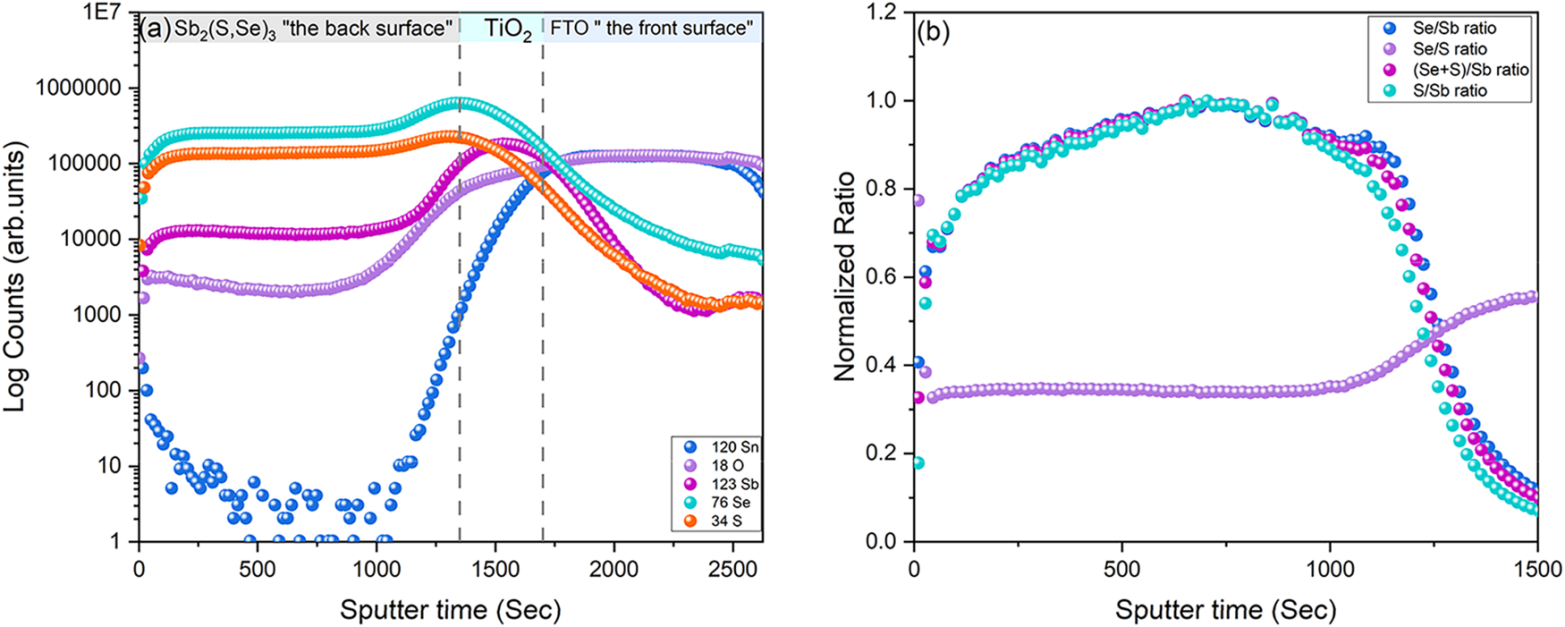
(a) SIMS depth profile of Sb_2_(S,Se)_3_ deposited by CSS on an FTO/TiO_2_ substrate with (b) normalized ratio of determined anion-to-cation and sulfur-to-selenium ratios. Approximate positions of device layers are marked on (a) as a guide.

**Figure 2:**
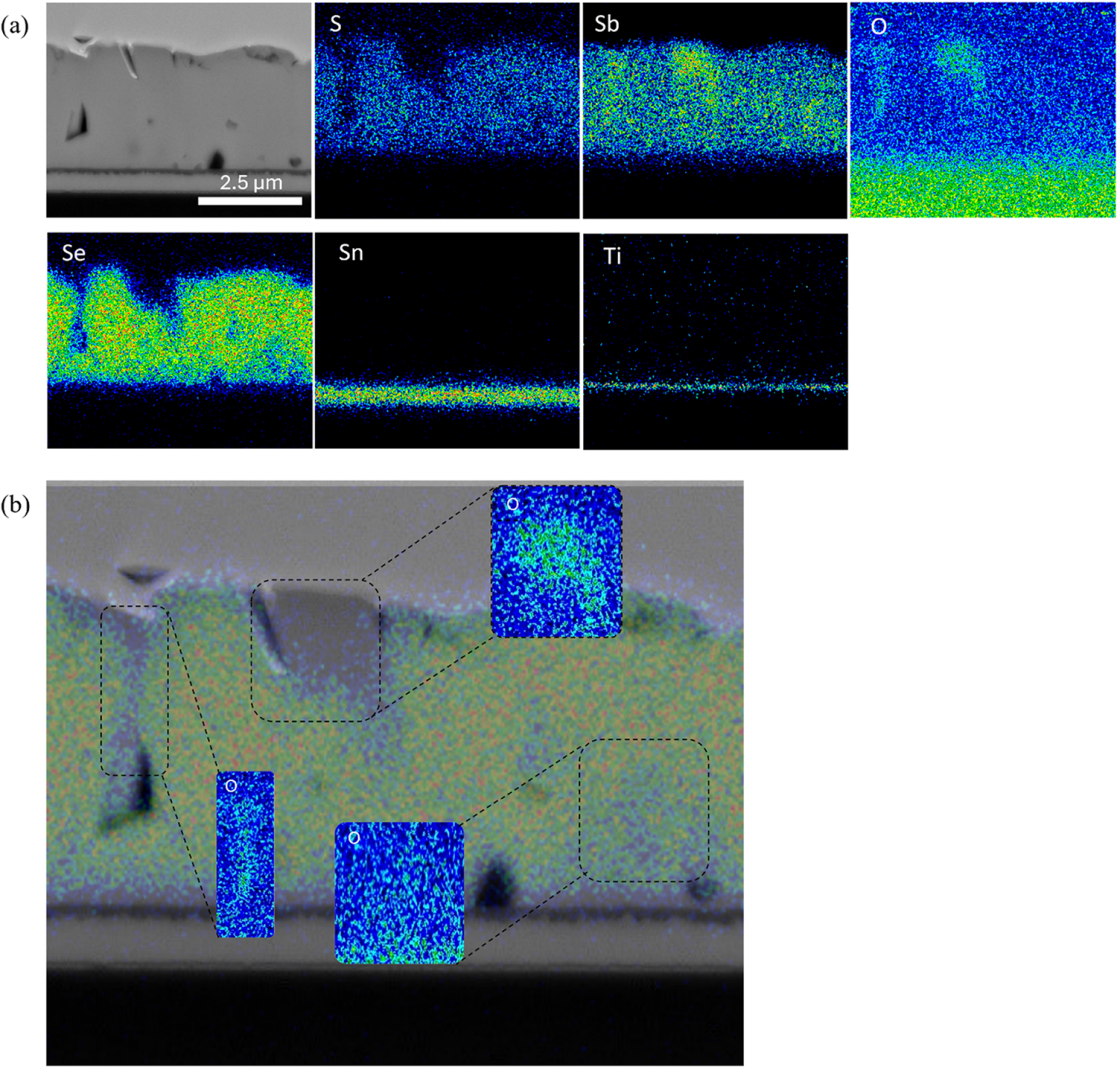
TEM analysis of FTO/TiO_2_/Sb_2_(S,Se)_3_ cross section showing (a) secondary electron image with associated EDS element intensity profiles and (b) secondary electron image with Se and S profiles overlaid and insets showing the O signal to highlight oxide-rich regions.

Figure 1a shows SIMS profiling using Cs^+^ ion sputtering of the sample with elemental spectra shown as sputter time versus count rate for Sn, O, Sb, Se, and S. The sample is probed starting from the free back surface, with approximate layer positions of the FTO/TiO_2_/Sb_2_(S,Se)_3_ sample structure shown for reference. These layer positions were determined from peaks in Ti and Sn signals, although exact interface positions are difficult to determine due to peak broadening effects. Using this data, the antimony to chalcogenide ratio values within the absorber region were normalized and are replotted as a function of sputter time (0–1300 s) in Figure 1b. This is done to assess the qualitative uniformity of the film composition rather than as absolute values due to the lack of available material calibration standards. In the bulk of the absorber region (∼50–1200 s sputter time), there is a large S signal, and the Se/S ratio is consistent throughout the absorber. This implies that the material has a relatively high and uniform sulfur content throughout the film. The ratios of Se/Sb, S/Sb, and (Se + S)/Sb are nearly identical, again implying a consistent Se/S content. All these ratio plots show a similar decrease in anion-to-cation ratio near the TiO_2_ interface and at the back surface; however, care must be taken not to overinterpret such count variations at such interface regions. In SIMS analysis signal at the very near back surface (i.e., during first few seconds of sputtering) is often susceptible to apparent composition variation, an artifact caused by variation of sputtering rate at the surface compared to bulk.^24^ Similarly, due to roughness effects, the apparent composition can change near interfaces due to an effective peak broadening. Even taking this into account, a gradual increase in anion/Sb ratios between 0 and 750 s in Figure 1b implies that the material may be slightly Sb-rich toward the back surface. However, it should be noted that the Sb signal remains relatively constant over the measured depth range; given the discrepancy in magnitude of the counts between this and anion signals, the stoichiometry variation this implies is likely small. There is a gradual increase in the oxygen content toward the back surface, which could further imply the formation of Sb_2_O_3_ or similar oxide phases. The presence of oxygen is, to an extent, anticipated due to the relatively low vacuum conditions of CSS and, more specifically, the use of nitrogen processing gas during growth. The gas is never entirely oxygen-free, so some contamination is expected, and while it has been suggested oxygen may act to passivate *V*_Se_ defects,^25^ in excess, the formation of oxide phases can be problematic due to increased resistivity.^26^

A device cross section was prepared via focused ion beam (FIB) lamella cutting and then analyzed via TEM with EDS to probe the lateral composition variations. Figure 2a shows a secondary electron image of a typical cross-sectional region, along with elemental S, Sb, O, Se, Sn, and Ti profiles. The positions of the Sn and Ti profiles clearly identify the positions of the TiO_2_ and FTO layers as reference points. Similar to the SIMS data, the S/Se signal remains relatively constant from front to back, with no noticeable separation regions of Se or S only. Notably, there are a number of distinctly chalcogenide-deficient regions, most clearly visible as hotspots in the Sb map and highlighted via a color overlay of Sb and S maps in Figure 2b. These regions also coincide with an increased oxygen signal (Figure 2b, inset), suggesting the formation of the Sb_2_O_3_ phase, although the presence of some content of metallic Sb cannot be discounted. The Sb-rich regions are also located adjacent to the formation of voids within the film. Because of the higher melting point of the oxide phase,^27^ formation of oxides will likely modify the grain growth and coalescence process of the film, which results in such voids. The mechanism here seems to be that some S or Se deficiency is compensated for with oxygen during the growth process, and this has a deleterious impact on grain structure. Despite this identified issue, given the overall quality of the film structure, it was decided to proceed with cell fabrication to allow device-level testing of the performance of the Sb_2_(S,Se)_3_.

After the preliminary study established the basic viability of the deposition method, a series of test cells with variation in deposition time from 1 to 15 min, correlating to a thickness range of 1.4–5.9 μm, were produced. Figure 3a–e shows the back surface SEM images from the device series. As one would expect from the grain coarsening process, the grain size increased for greater film thickness, ranging from ∼1.3 to ∼4 μm grain diameter in the thickest film. All films displayed good coverage, free from clearly visible pinholes and with a well-defined grain structure. The films intermittently show the presence of some grains with an atypical but distinct pyramidal shape, as shown in Figure 1b. This tetragonal shape has been observed in our previous work on Sb_2_Se_3_ films deposited by CSS and identified as the Sb_2_O_3_ oxide phase,^28^ and similarly, here energy-dispersive spectroscopy (EDS) analysis of the pyramidal grains gave a >50 atom % oxygen content (Figure S1). These grains are therefore presumed to be Sb_2_O_3_, and their presence is consistent with slight chalcogenide reduction at the near back surface in SIMS analysis.

**Figure 3:**
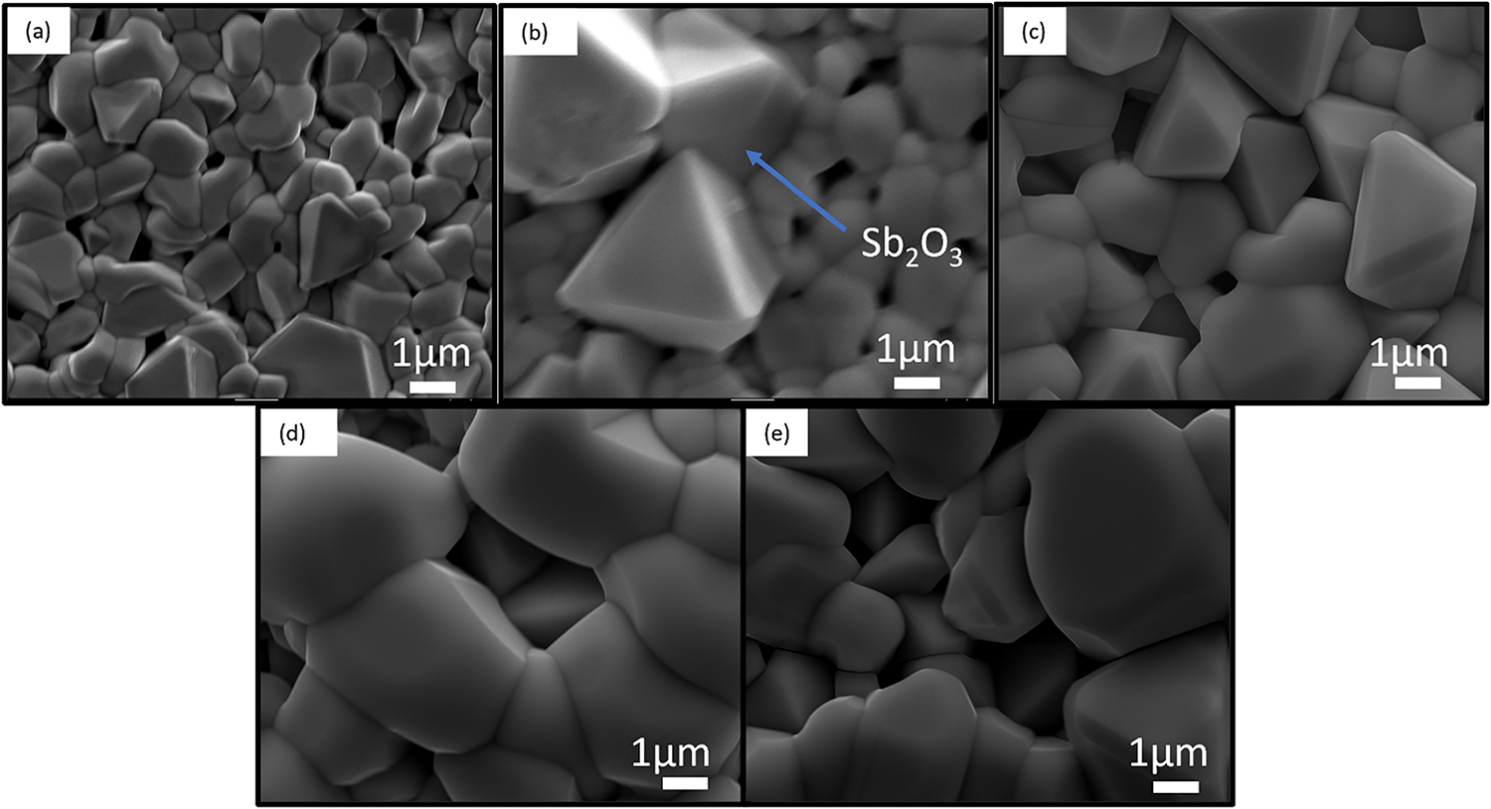
SEM images of Sb_2_(S,Se)_3_ deposited by CSS for (a) 1 min, (b) 2 min, (c) 5 min, (d) 10 min, and (e) 15 min.

Figure 4 shows the bandgap determination from the optical absorption measurements. The bandgap of Sb_2_(S,Se)_3_ determined ranges between 1.30 and 1. 35 eV; however, for growth times ≤ 10 min, the bandgap value is consistent in the 1.34–1.35 eV range. The slight variation of the bandgap with film thickness likely results from variations in absorption strength, which Tauc analysis struggles to differentiate. This is significantly higher than that of Sb_2_Se_3_ (1.18 eV),^29^ but lower than anticipated, given a 50:50 sulfur-to-selenium ratio in the fabricated source material for which we would expect ∼1.4 eV. This would imply a sulfur fraction closer to 0.4 and that some sulfur loss has occurred during the deposition process.

**Figure 4:**
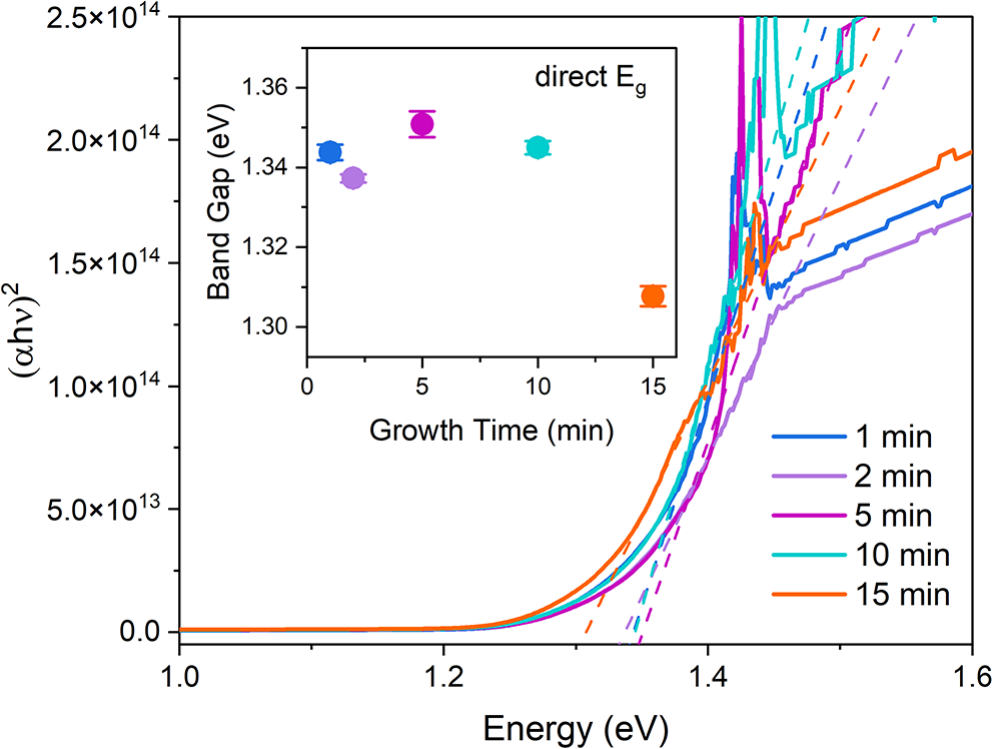
Tauc plot for a direct bandgap (*n* = 2) analysis of Sb_2_(S,Se)_3_ device stacks with various deposition times and determined bandgap in the inset.

Figure 5a shows XRD analysis of the same sample series along with an Sb_2_Se_3_ reference pattern (reference patterns for the S/Se phase being unavailable), with the specific peaks identified in 3 different ranges: (a) 11–17°, (b) 26–31°, and (c) 26–31.5°. Notably, it is difficult to distinguish between the Sb_2_(S,Se)_3_ and Sb_2_O_3_ due to the overlapping peaks resulting from the similar but nonidentical lattice constants in the a and b directions, as well as the uncertainty in the exact Sb_2_(S,Se)_3_ peak positions. For that reason, additional GIXRD measurements were performed for specific peaks to distinguish between the two, and Figure 5a shows a peak at 13.7°, which can be reliably assigned to Sb_2_O_3_, since it is well spaced from those of Sb_2_(S,Se)_3_. This peak displays a strong decrease in the intensity with probing deeper in the film, which is consistent with SIMS analysis, suggesting more oxygen at the back surface, and gives us a way to distinguish between Sb_2_(S,Se)_3_ and Sb_2_O_3_ peaks. For example, Figure 5c shows two peaks, at 27.6 and 28.5°. We are able to relate the lower-angle peak to Sb_2_O_3_ since the signal decreases for higher incident angles (i.e., increased penetration depth), as shown above. Conversely, the peak at 28.5° corresponds to a Bragg reflection associated with the (221) planes. The peak shows a shift to a higher 2θ angle relative to the Sb_2_Se_3_, as would be expected for a Sb_2_(S,Se)_3_ alloy, which allows us to make an estimate for the sulfur content in the films. The 2θ shift was determined to be 0.18°, which equates to a Sb_2_(S,Se)_3_ film with a sulfur fraction of ∼0.22 according to Vegard’s law, less than the fraction inferred from the optical analysis. All samples showed an equivalent shift, indicating the S/Se content is consistent. We would note that estimates of alloy composition based on Vegard’s law should be interpreted with caution in thin films, where strain and defects can shift peak positions. Nonetheless, the peak shift to a lower angle compared to the powdered material qualitatively agrees with EDX, SIMS, and optical analysis, suggesting sulfur loss during CSS deposition.

**Figure 5:**
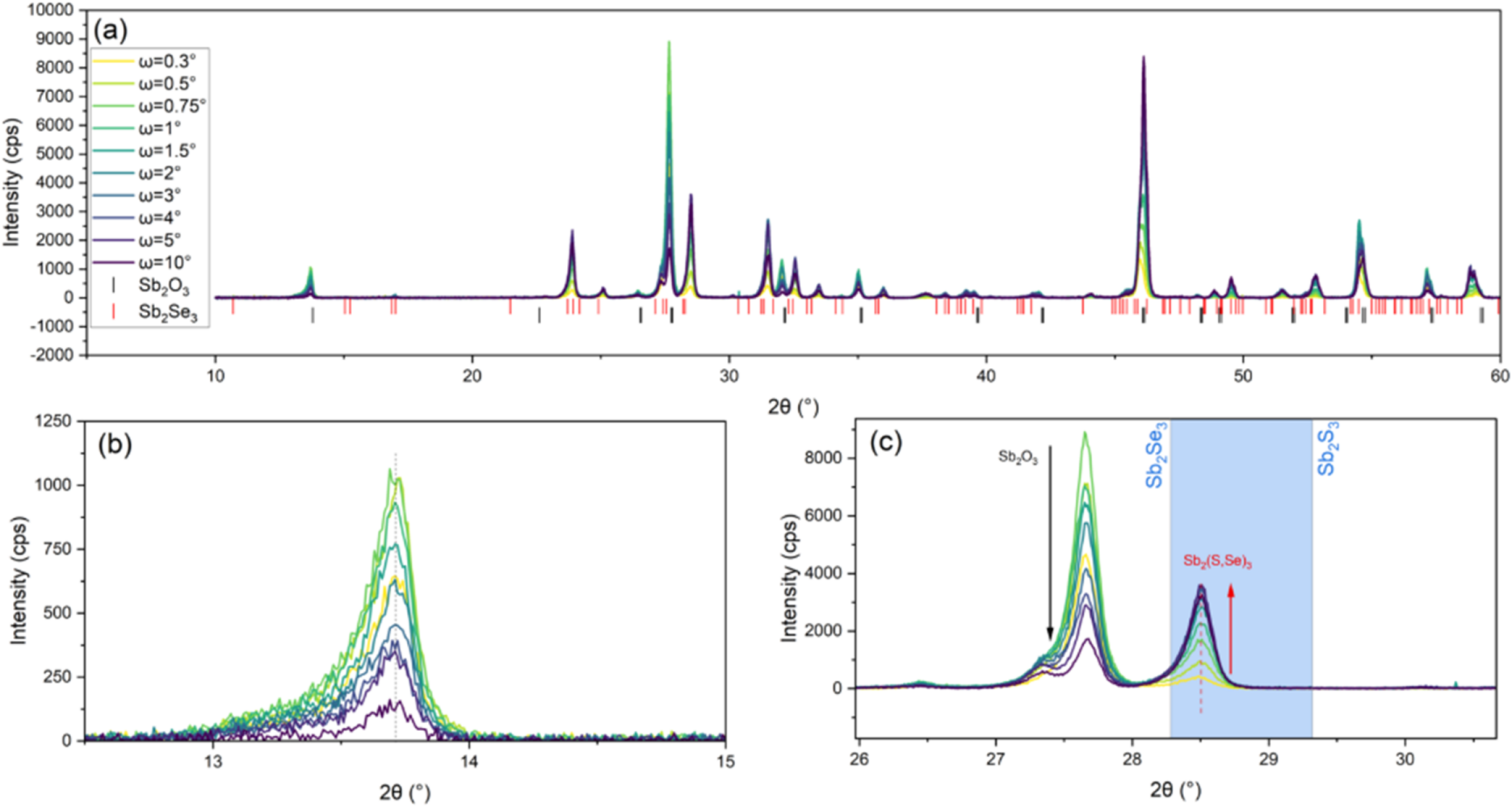
(a) XRD patterns recorded for TiO_2_/Sb_2_(S,Se)_3_ with different deposition times with Sb_2_Se_3_ and Sb_2_O_3_ reference patterns, (b) a large view of the XRD pattern from 12.5 to 15°, and (c) a large view of the XRD pattern from 26 to 30.5°.

The presence of Sb_2_O_3_ was further confirmed via Raman analysis. Multiple regions were scanned, focusing either on the bare absorber surface or on the distinctly pyramidal grains previously identified as Sb_2_O_3_.^28^ As shown below, the Raman spectra from the bare absorber surface exhibit the characteristic peaks expected for Sb_2_(S,Se)_3_, dominated by a strong peak at around 191 cm^–1^. There is only a minor contribution at 155 cm^–1^, supporting the XRD findings showing a well-oriented texture with minimal (*hk*0) orientation.^30^ Additional weak peaks observed above 250 cm^–1^ are attributed either to surface oxidation or subsurface oxide impurities, as previously seen in cross-sectional SEM analysis (Figure 3).

In contrast, Raman spectra collected from the pyramidal regions (Figure 6) display sharper and more intense peaks corresponding to Sb_2_O_3_ with dominant features around 187 and 253 cm^–1^. These peaks are commonly reported as indicators of secondary phases in Sb_2_Se_3_ films,^31^ and the near-exclusive appearance of the oxide-related peaks in this scan indicates that the Sb_2_O_3_ impurity is highly localized.

**Figure 6:**
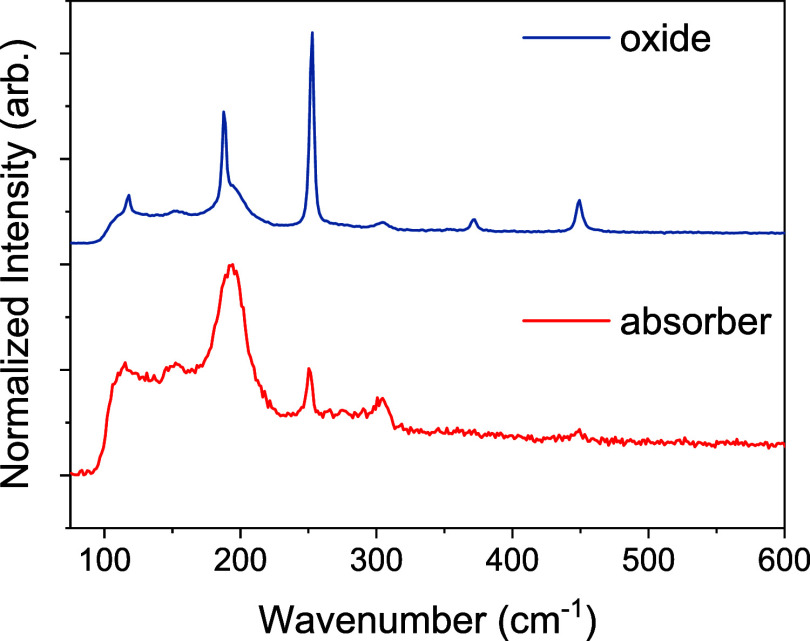
Raman spectra collected from different regions of the film surface corresponding to the bare absorber (bottom) and darker regions with visible oxide-related pyramidal grains (top).

To further investigate the spatial distribution of the oxide phase, a 20 μm × 22 μm area was mapped across the sample surface, with Raman spectra acquired every 2 μm, as shown in Figure 7. Intensity mapping of the 252, 372, and 449 cm^–1^ peaks confirms the segregation of the Sb_2_O_3_ phase at discrete locations, spatially coinciding with the pyramidal grains.

**Figure 7:**
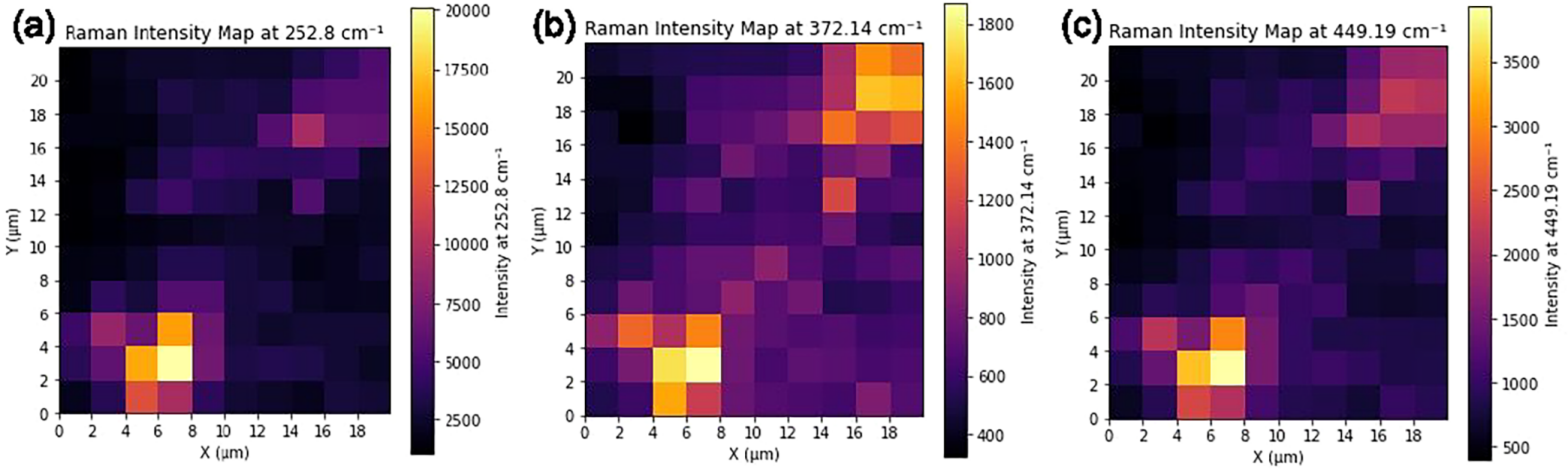
Heatmaps showing the spatial variation in the intensity of Raman peaks at (a) 252.8, (b) 372.14, and (c) 449.19 cm^–1^, indicating the presence of Sb_2_O_3_ across a 20 μm × 22 μm grid, with data taken at 2 μm intervals.

Comparing this result with our previous work on Sb_2_Se_3_ deposited using by CSS under a variety of conditions,^28^ we observe several hkl peaks, such as 130, 041, 141, and 061, which strongly appear in previous samples but are absent in the samples from this study.

This XRD analysis provides similar observations to those of EDS and SIMS analysis. Again, it suggests some S loss during CSS deposition, which leads to Sb-rich phases, which then allow for oxide formation. Because these phases form preferentially toward the near back surface, it is also possible that oxide formation occurs post-deposition. Some Se loss may similarly be occurring, but given the lower than expected sulfur content determined, and the fact this has not been observed to this extent in prior work on Sb_2_Se_3_, sulfur loss seems the more likely explanation.

Complete device structures were then fabricated via the addition of a P3HT HTL and back contacts to assess the device performance relative to our typical Sb_2_Se_3_ platform^28^ via *J*–*V* analysis. Figure 8a–d shows box plot analysis for performance parameters of all contacts measured as a function of the deposition time with parameters for highest efficiency contacts from each in Table 1. Associated *J*–*V* and EQE curves are given in Figure 9. The highest efficiency device was for the shortest deposition time, with a short-circuit current density *J*_sc_ of 25.6 mA cm^–2^, open-circuit voltage *V*_oc_ of 0.49 V, fill factor FF of 33.6%, and η of 4.3%. As the deposition time increased, the efficiency decreased to a peak of 1.6%, through reductions in both the *J*_sc_ and FF. This is likely due to the film becoming overly thick, but the presence of oxide phases potentially adds an additional series resistance component, meaning *R*_s_ values are high for all samples compared Sb_2_Se_3_.^28^ In contrast, there was a reduced *R*_sh_ for thicker films causing further reduction of the FF, a trend that is clearly evident in the *J*–*V* curve in Figure 9a. The shunt and series resistances were estimated from the gradient of the *J*–*V* curves in reverse bias and at *V*_oc_, respectively. While there is some decrease, the *V*_oc_ does not follow as consistent a trend with longer deposition times as the other parameters, but the highest *V*_oc_ is still achieved with the shortest deposition time. Comparing the highest *V*_oc_ value of 0.49 V for Sb_2_(S,Se)_3_ with our previous Sb_2_Se_3_ result of 0.43 V,^28^ we observe a significant improvement of ∼60 mV. Open-circuit voltage is presumed to be determined primarily by the absorber bandgap, with a larger bandgap generally leading to a higher *V*_oc_. Hence, the improvement here can be attributed to the wider bandgap in Sb_2_(S,Se)_3_. EQE curves give us an additional opportunity to make an assessment of the bandgap. As noted in Alomora et al.,^23^ *E*_g_ determined from EQE, which they term as *E*_g,pv_, characterizes the occupied density of states in the full device as opposed to the isolated absorber material. It thereby accounts for excitonic and thermal broadening contributions as well as the film thickness and therefore gives a better bandgap approximation to relate to the detailed balance limit. Figure 9b shows EQE curves for this device series, along with EQE derivative plots in Figure 9c and the bandgap values extracted from this in Figure 9d. This is essentially a way to assess the position of long wavelength cutoff from EQE and a mechanism to estimate the effective bandgap. Here, the device bandgaps are 1.37–1.42 eV higher than those determined by optical analysis and closer to that expected for the source material composition. For comparison determined using the same EQE analysis for a similar Sb_2_Se_3_ device, the bandgap is 1.2 eV,^22^ in comparison to an optically determined bandgap of 1.18 eV.^29^ Hence, we see a similar degree of shift for the optically and electronically determined bandgaps for Sb_2_(S,Se)_3_. The bandgap determined here would imply that there has been fairly minimal sulfur loss; however, the specific level is difficult to discern. Given the observations from comparative XRD, SIMS, and EDX analysis, it seems that, while there is clearly a high sulfur content retained in the film, some loss does occur leading to the formation of oxide phases. Routes to minimize the degree and progression of this sulfur loss (i.e., does source material continually degrade with use) and removal of discrete oxide or chalcogenide-poor regions is a clear area for the next steps in the development of this approach.

**Table 1:** PV Parameters Performance for the Cells

growth layer time	*V*_OC_ [V]	*J*_SC_ [mA cm^–2^]	FF [%]	η [%]	*R*_S_ [Ω cm^–2^]	*R*_SH_ [Ω cm^–2^]
1 min	0.49	25.6	33.6	4.3	11.3	343
2 min	0.48	20.2	36.8	3.5	12	182
5 min	0.45	15.6	29.7	2.1	21.6	144.3
10 min	0.44	13.8	29.1	1.8	25.1	116.6
15 min	0.48	12.9	26	1.6	34.2	98.7

**Figure 8:**
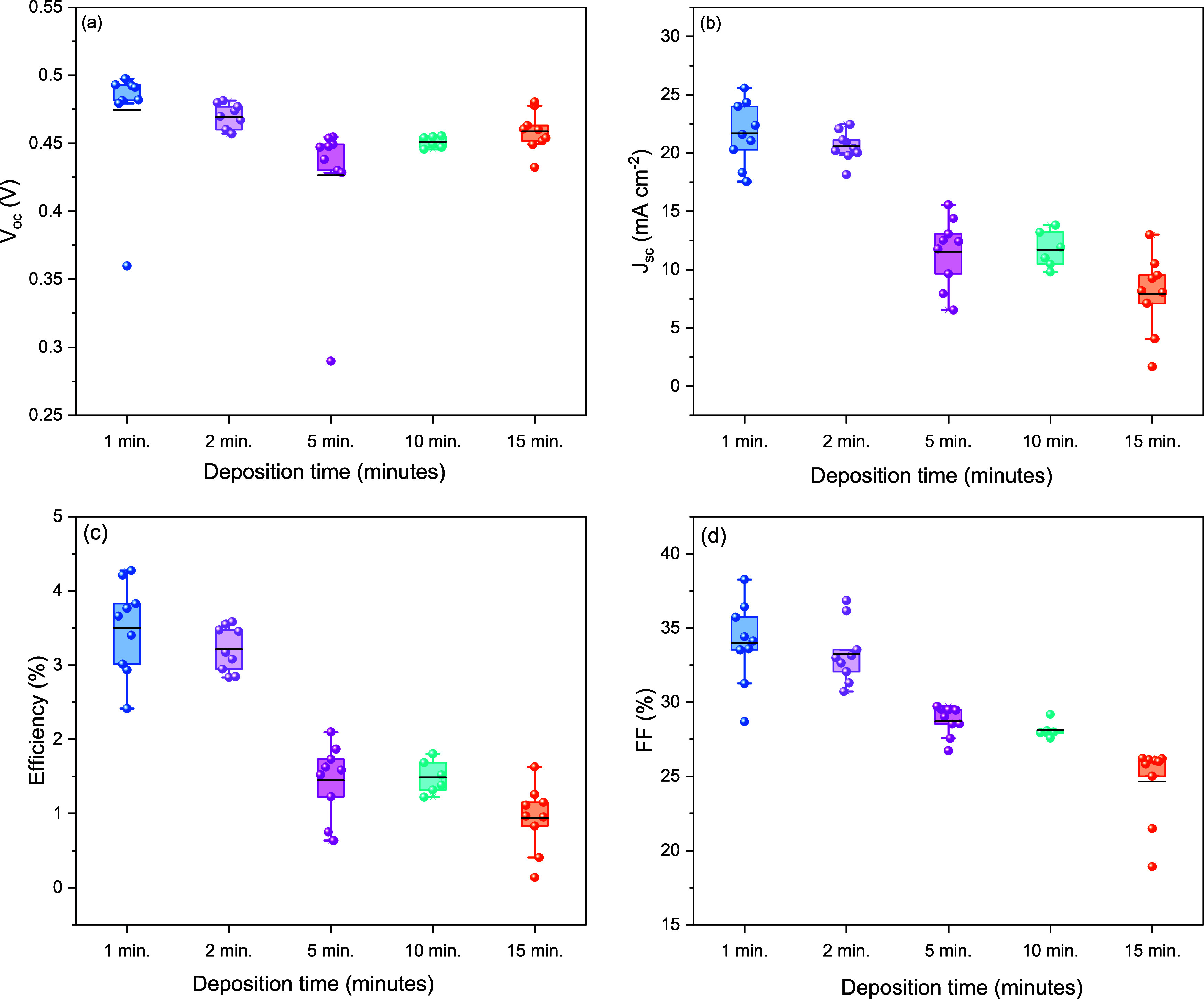
Performance parameters for fabricated Sb_2_(S,Se)_3_ devices as a function of deposition time extracted from *J*–*V* curves showing (a) open-circuit voltage, (b) short-circuit current density, (c) efficiency, and (d) fill factor.

**Figure 9:**
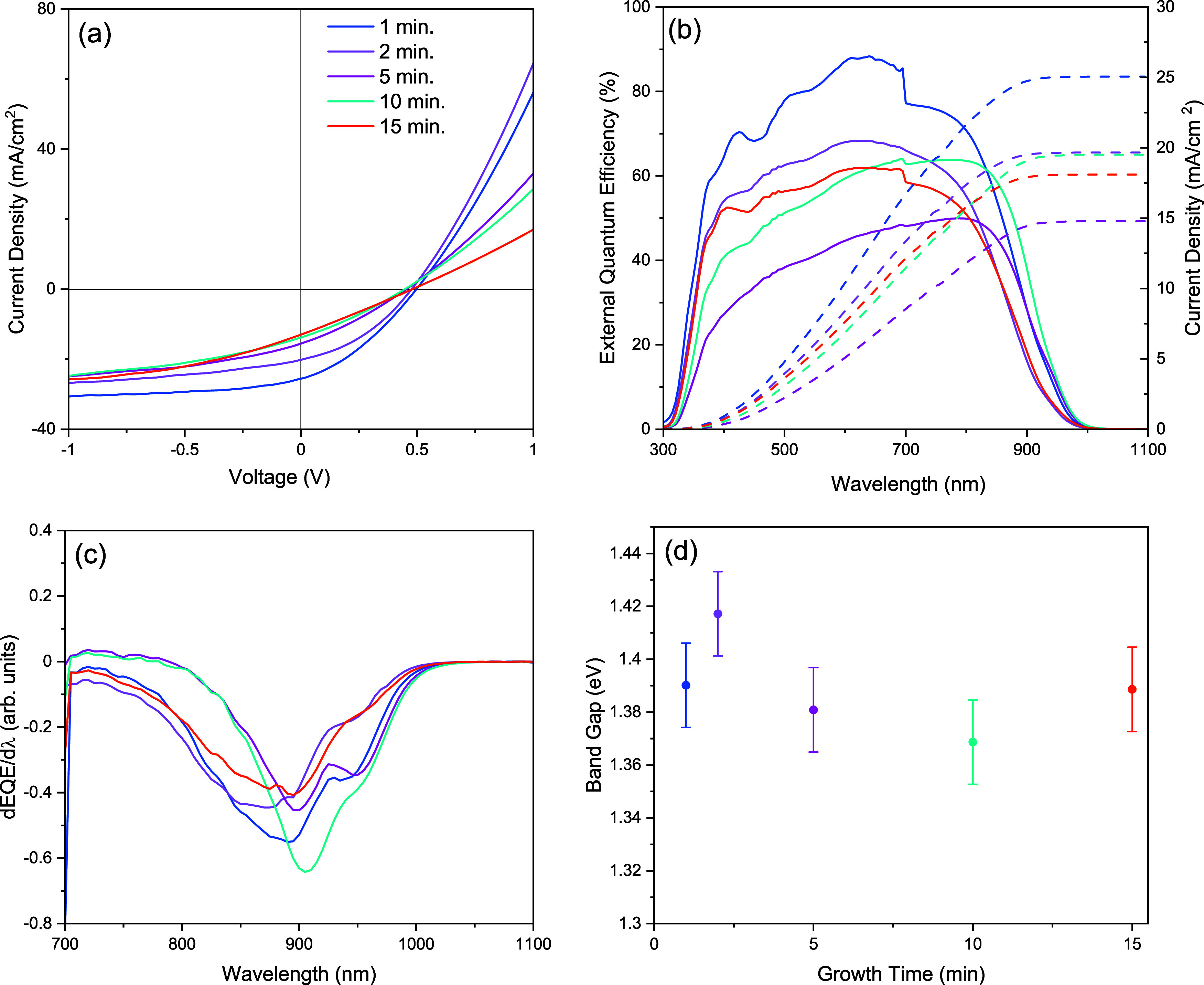
(a) *J*–*V* and (b) EQE curves of highest efficiency contacts for Sb_2_(S,Se)_3_ devices with various deposition times. (c) EQE Derivative curves for same contacts and (d) extracted bandgap.

## 4 Conclusions

This work demonstrates an approach for Sb_2_(S,Se)_3_ deposition via a CSS process from a single source. Our results indicate that while this approach is reasonably effective, leading to devices with efficiency of over 4%, there are clear issues with sulfur loss during the deposition process, resulting in Sb-rich material and the formation of oxide phases. This subsequently leads to distinct oxide regions within the film interior and the formation of adjacent voids as a result. While this is attributed here to sulfur loss, it is not ruled out that selenium loss may also occur and therefore of wider consideration as a contributing factor oxide formation during a Sb_2_Se_3_ CSS process. As noted earlier, we have previously identified the formation of undesirable oxide phases^28^ in the selenide, as well as the formation of similar voids within the material.^32^ While subtle shifts in stoichiometry are notoriously difficult to identify, it is worth considering if chalcogenide loss in antimony chalcogenides is a more significant problem overall. Correction of this chalcogenide loss may offer a potential route to performance improvement for both the selenide and sulfoselenide materials. Future work will be needed to identify the impact of modification of the overall sulfur in the CSS source material along with a direct comparison with chalcogenide loss in the binary sulfide and selenide material. Lower deposition temperatures seem an obvious future strategy to minimize sulfur loss during deposition by minimizing the possibility of source decomposition. Additional postgrowth sulfurization process may also be beneficial, but such approaches would not limit oxide formation during the deposition process. Instead, methods to preserve the desired composition throughout the deposition process should be sought. This study has however demonstrated a route to fabricate Sb_2_(S,Se)_3_ from a single CSS source, while also identifying key issues with the process. This approach does offer promise though due to the increase in *V*_oc_ compared to the equivalent Sb_2_Se_3_ device, if stoichiometry issues can be overcome.

## Supplementary Material



## Data Availability

Data supporting this work are available from DOI: 10.5281/zenodo.17287969.

## Notes

EDS of TiO_2_/Sb_2_(S,Se)_3_ sample (Figure S1) and EDS of TiO_2_/Sb_2_(S,Se)_3_ sample (Figure S2) (PDF)
