# Supplementary material for: Close Space Sublimation Growth of Sb2(S,Se)3 Thin-Film Solar Cells
Source: ACS Appl Mater Interfaces. 2025 Oct 8;17(42):58398–407. doi: 10.1021/acsami.5c10627 (PMC12557190; doi:10.1021/acsami.5c10627)
Supplement: Supplementary file 1 [file am5c10627_si_001.pdf]

Supporting Information

Close Space Sublimation Growth of $Sb_2(S,Se)_3$ Thin Film Solar Cells

Daniya A. Sindi[1,2], Thomas P. Shalvey[1], Matthew. J. Smiles[1], Tim. D. Veal[1], Leon Bowen[3] and Jonathan. D. Major[1*]

1 - Department of Physics at Stephenson Inst. Renewable Energy, University of Liverpool, Liverpool, L69 7ZF, UK

2 - Department of Physics, College of Science, Umm Al-Qura University, Makkah, 24382, Saudi Arabia

3 - Department of Physics, G.J. Russell Microscopy Facility, Durham University, Durham, DH1 3LE, UK

*corresponding author email: jonmajor@liverpool.ac.uk

(a)

Element	Signal Type	Atomic %
O	EDS	53.98
S	EDS	5.89
Se	EDS	14.89
Sb	EDS	25.24
Total		100.00

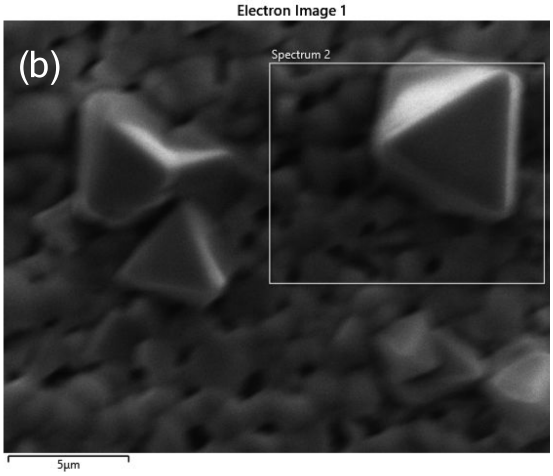

Figure S1: EDS atomic ratio for $TiO_2/Sb_2(S,Se)_3$ film element confirm oxygen for (a-b) the pyramidal grains.

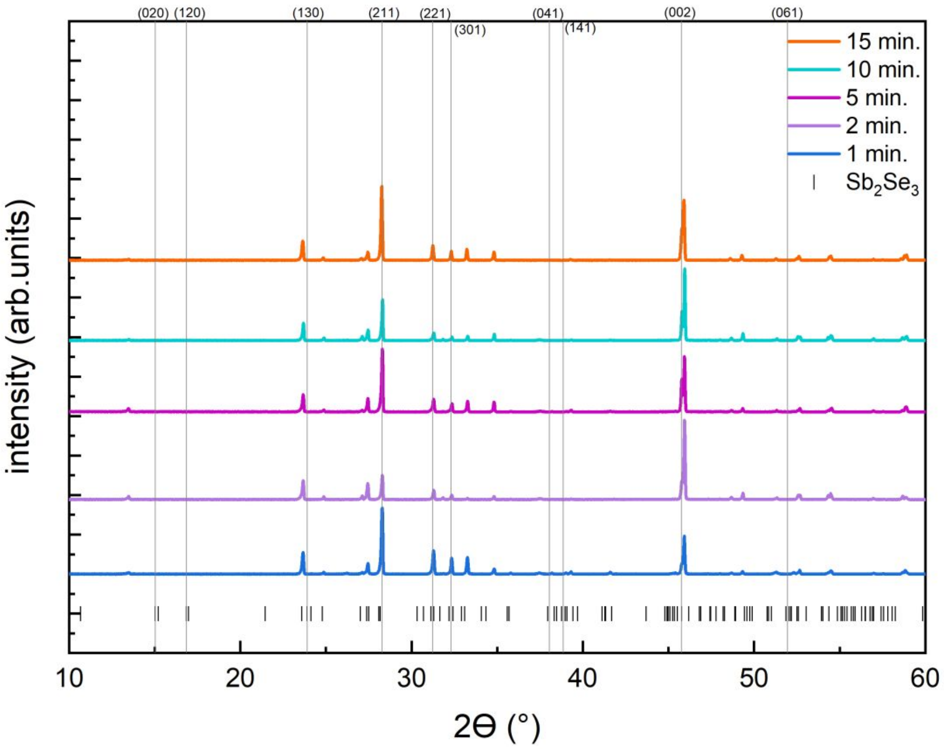

Figure S2: XRD patterns recorded for $TiO_2/Sb_2(S,Se)_3$ films
